# A sublimation heat engine

**DOI:** 10.1038/ncomms7390

**Published:** 2015-03-03

**Authors:** Gary G. Wells, Rodrigo Ledesma-Aguilar, Glen McHale, Khellil Sefiane

**Affiliations:** 1 Department of Physics and Electrical Engineering, Northumbria University, Ellison Place, Newcastle upon Tyne NE1 8ST, UK; 2 School of Engineering, University of Edinburgh, The King’s Buildings, Mayfield Road, Scotland EH9 3FB, UK; 3 International Institute for Carbon-Neutral Energy Research (I2CNER) and Department of Mechanical Engineering, Kyushu University, 744 Motooka, Nishi-ku Fukuoka, Fukuoka 819-0395, Japan

## Abstract

Heat engines are based on the physical realization of a thermodynamic cycle, most famously the liquid–vapour Rankine cycle used for steam engines. Here we present a sublimation heat engine, which can convert temperature differences into mechanical work via the Leidenfrost effect. Through controlled experiments, quantified by a hydrodynamic model, we show that levitating dry-ice blocks rotate on hot turbine-like surfaces at a rate controlled by the turbine geometry, temperature difference and solid material properties. The rotational motion of the dry-ice loads is converted into electric power by coupling to a magnetic coil system. We extend our concept to liquid loads, generalizing the realization of the new engine to both sublimation and the instantaneous vapourization of liquids. Our results support the feasibility of low-friction *in situ* energy harvesting from both liquids and ices. Our concept is potentially relevant in challenging situations such as deep drilling, outer space exploration or micro-mechanical manipulation.

## Abstract (web summary)

Heat engines are designed to convert thermal energy into mechanical work through a thermodynamic cycle. Here, Wells *et al*. show a cycle based on a sublimation process, where a disk of dry ice that rotates on a hot surface due to the Leidenfrost effect is coupled to a simple electromagnetic generator.

## Graphical abstract

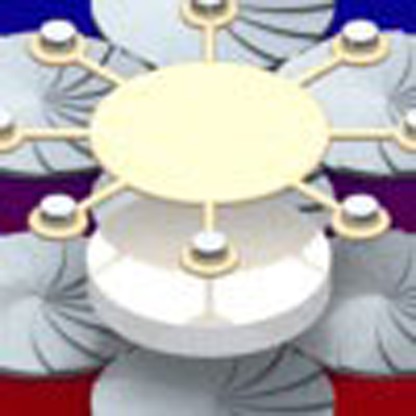

Ever since the invention of water mills in antiquity, through the development of the steam engine in the 18th century, and up to today’s turbines, many power generators rely on the principle of harnessing a fluid stream to drive rotational motion. Whether it is to power a wind farm or a micro electromechanical system, a central challenge remains the effective conversion of fluid-flow energy into useful work despite the friction of a bearing^1^,^2^. Heat engines add many practical advantages to energy conversion, most notably the ability to convert the stored chemical energy of fuels, such as coal, gas or radioactive materials, into heat and eventually into mechanical work. However, standard engines often involve several steps, each decreasing the efficiency, with particular care needed to minimize friction when a rotating turbine is involved.

Leidenfrost^3^ first noticed the remarkable low-friction properties of the instantaneous vapourization of a liquid, also known as thin-film boiling, in his 1756 ‘tract about some common properties of water’, reporting that a small speck of dust trapped at the interface of a levitating droplet would move ‘with a wonderful velocity’. More recently, it has been shown that effective directed motion of Leidenfrost drops and solids can be achieved by rectifying the flow within their supporting vapour layer using hot anisotropic ratchets^4^,^5^,^6^,^7^. Because the driving force is supplied by underlying vapour, the resistance experienced by these Leidenfrost ‘karts’ is very low^8^.

Harvesting thermal energy using sublimation as a phase-change mechanism via the Leidenfrost effect is an attractive concept, as it offers the key advantage of a virtually friction-free bearing provided by the vapour layer. In addition, alternative, non-traditional fuels can be used to circumvent the complications posed by extreme temperature and pressure conditions of exotic landscapes. For example, it has been recently suggested that, for deep space applications, locally available resources (ices of H_2_O, CO_2_ and CH_4_) on the surfaces of planetary bodies could be sources for use in sublimation^9^. The abundance of such resources is highlighted by recent reports of ‘linear gullies on Mars’ carved by slabs of solid CO_2_ sliding down inclines. Such a process is thought to occur as a consequence of seasonal variations in the environmental temperature, which drive the sublimation of dry-ice deposits^10^. This highlights that low pressures and high temperature differences naturally occurring in exotic environments could make energy harvesting and power generation based on alternative heat cycles, and using locally available ices, feasible.

In this paper, we present a sublimation heat engine that exploits the Leidenfrost effect to convert temperature differences into rotational motion. Our concept relies on Leidenfrost vapour rectification by turbine-like surfaces to create low-friction suspended rotors, and is both applicable to sublimating solids (dry ice) and vapourizing liquids (water). Our experiments focus on the effect of the driving temperature difference, load size and turbine geometry. We further rationalize our results by deriving a creeping flow hydrodynamic model, obtaining an excellent agreement with the experiments. We also build a simple magnetic coil generator based on a dry-ice Leidenfrost rotor, thus providing a proof-of-concept of our method as a new means of energy harvesting.

## Results

### Heat engine concept

**Figure 1:**
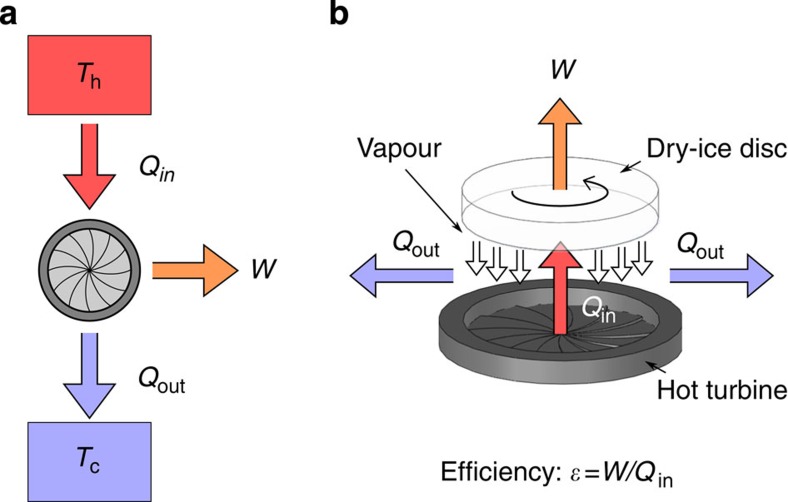
Concept of a Leidenfrost heat engine. (**a**) A heat engine operates between two reservoirs held at temperatures *T*_h_ and *T*_c_, where *T*_h_>*T*_c_. The engine converts part of the heat absorbed, *Q*_in_, into work, *W*. The remaining energy, *Q*_out_, is dissipated to the surroundings. (**b**) A Leidenfrost engine uses a hot turbine whose temperature, *T*_h_, is held above the Leidenfrost point of a disc of dry ice. The excess pressure sustains the levitation of the disc. The released vapour is rectified by the underlying turbine, driving the rotation of the disc and generates mechanical work.

The general concept of a heat engine, depicted in Fig. 1a, is centred on a working substance that absorbs a quantity of heat *Q*_in_ from a hot reservoir, held at temperature *T*_h_. Part of the heat absorbed is converted into work *W*, while a quantity *Q*_out_ is dissipated to a cooler reservoir held at temperature *T*_c_. The underpinning basis of our heat engine is the achievement of Leidenfrost-based rotational motion, which we depict in Fig. 1b. The working substance, in the present case, solid CO_2_ or liquid H_2_O, is converted into superheated vapour by absorbing a quantity of heat *Q*_in_ supplied by a neighbouring turbine-like surface held at a temperature *T*_c_>*T*_L_, where *T*_L_ is the temperature of the Leidenfrost point. The released vapour is then rectified to produce mechanical work, *W*, and cooled to the original temperature *T*_c_, giving off an amount *Q*_out_ of heat to the surroundings. This new thermal cycle is the solid-to-vapour analogue of the liquid-to-vapour Rankine cycle, which is widely used in steam-powered engines. However, the present cycle involves sublimation (or thin-film boiling) as the phase change and ensures the stabilization of a low-friction vapour layer by keeping the temperature of the hot surface above *T*_L_. The first quantity of interest is the theoretical thermal efficiency of the engine. This is the maximum efficiency attainable in the absence of operational losses. The theoretical efficiency, *ε*≡1–*Q*_in_/*Q*_out_, is limited by the efficiency of a Carnot engine operating between the two same heat reservoirs, *ε*≡1–*T*_c_/*T*_h_. One approximation of the theoretical efficiency of the Leidenfrost engine, motivated by the approach used in a simplified Rankine cycle, is *ε*≈1–*T*_c_/*T*_ave_, where *T*_ave_ is the average temperature between the temperature of the working substance and the superheating temperature after the phase change. A more precise approximation would depend on the specific thermodynamic phase diagram of the working substance^11^. For example, for dry ice taking *T*_ave_=(*T*_c_+*T*_h_)/2, with *T*_h_=500 °C and *T*_c_=−78 °C, gives a maximum thermal efficiency of *ε*≃0.67; such a high efficiency arises because of the high temperature differences involved.

### Experimental

**Figure 2:**
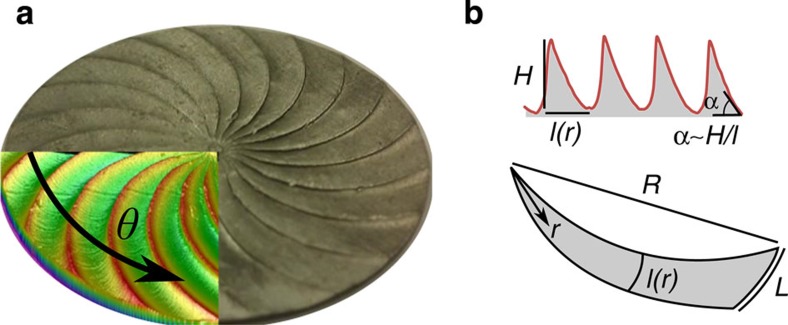
Turbine geometry. (**a**) Computer numerical control (CNC) machined aluminium turbine, composed of *N* asymmetric teeth (disc radius *R*=2 cm). Inset: colour-coded height profile. (**b**) Height profile along the angular coordinate, *θ* (top) and tooth geometry (bottom). The height of the teeth, *H*, is constant. For a single tooth (bottom panel), the local azimuthal length, *l*(*r*), increases radially outwards. Therefore, the tooth inclination angle decreases with increasing radial distance from the centre.

For our experiments, we fabricated aluminium turbine-like textured substrates of varying radius *R* with *N*=20 asymmetric teeth using standard computer numerical control machining (Fig. 2a). The surface of the turbines was characterized using surface profilometry (Fig. 2a inset). Figure 2b shows the height profile of the turbine at a fixed radius along the angular coordinate, *θ*. The surfaces were designed to keep the height of the ridges, *H*, constant with a sweep based on a standard axial gas turbine design. The local azimuthal length of the ridges, *l*, is determined by the number of teeth, *N*, and increases with increasing distance from the centre, *r*, that is, *l*(*r*)=2*πr*/*N*. Therefore, the local inclination angle of the teeth along the azimuthal direction, *θ*, decreases with increasing distance from the centre, *r*, according to tan^−1^(*H*/*l*)=tan^−1^(*R*/*r*tan*α*), where *α* is the inclination angle at the edge, where the length of the teeth reaches its maximum value, *L*=*l*(*R*).

**Figure 3:**
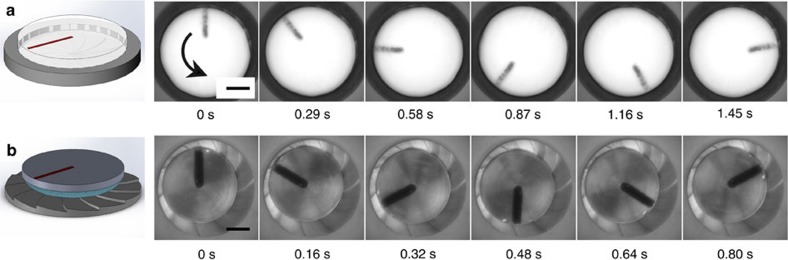
Geometry and time sequence of Leidenfrost-powered rotors. (**a**) A disc of dry ice (*R*=2 cm) is placed on a hot turbine-like surface (*N*=20, *α*=0.1 and *T*_h_≈500 °C). The panels show a sequence over time, showing the rotation of the disc. (**b**) A drop of water supporting a metal plate is placed on top of the turbine. The drop forms a film that wets the top plate. As shown in the time sequence, the drop and the top plate rotate as a single combined object on contact with the hot underlying surface in the counterclockwise direction, following the downhill variation of the turbine teeth. The length of the scale bars, 1 cm.

Solid CO_2_ discs were placed on top of the turbines as shown schematically in Fig. 3a. The turbines were pre-heated to temperatures in the range 350 °C<*T*<500 °C. We identified two distinctive regimes determined by the weight of the discs. For large weights, Leidenfrost-induced levitation is hampered by the underlying surface. In the experiments, this was evident by imprints left by the turbine on the surface of the dry-ice disc. Decreasing the mass of the loads below a critical value *m*_c_ leads to a second regime where the discs levitate freely on top of the turbine-like surface. However, a marked difference to the familiar Leidenfrost levitation is that the turbine-like substrates drive the rotation of the discs along the angular direction. Figure 3a shows a time sequence of the rotation of a 2.0 cm CO_2_ disc on top of a turbine held at a temperature *T*_h_=500 °C (see also Supplementary Movie 1). Because the substrates are fixed, the CO_2_ discs act as self-powered rotors. Stable rotation was achieved by using confining rings, which help redirect the vapour flow across the gap formed between the dry-ice disc and the ring walls. Therefore, it is reasonable to assume that the disc is kept in a centred position because of Bernoulli’s principle: a small displacement of the disc towards the boundary ring causes a higher pressure acting on the region closer to the ring, therefore displacing the disc back to the centre. We carried out a second set of experiments, under identical conditions, using water droplets in place of the CO_2_ discs. The droplets were stabilized by placing a hydrophilic metal plate on top of the droplet as shown in the schematic in Fig. 3b (see also Supplementary Movie 2). As with the CO_2_ discs, the Leidenfrost-induced thin-film boiling of the droplet results in rotational motion, in this case evident by the rotation of the top plate (Fig. 3b right panels). In both cases, rotation occurred in the downhill direction along the teeth of the turbine.

### Model of a turbine surface and Leidenfrost rotor

**Figure 4:**
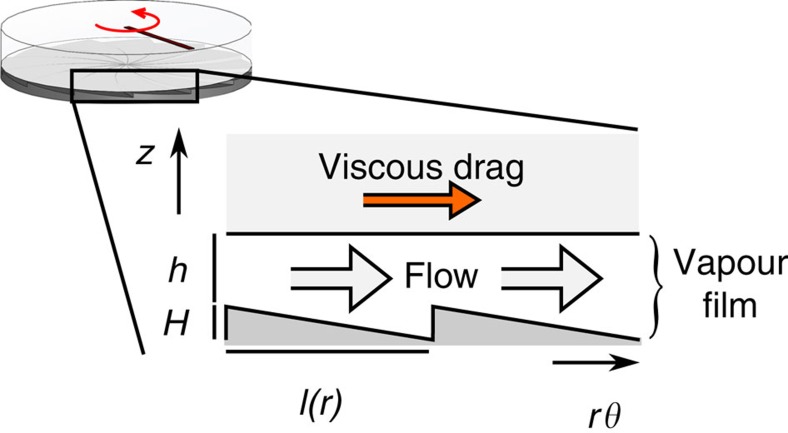
Rectification mechanism for Leidenfrost rotation. The vapour released by the CO_2_ disc or the water film creates a layer of thickness *h* between the surface of the turbine and the levitating surface. The underlying pattern drives the vapour flow downhill along the teeth. The resulting viscous drag drives the rotation of the levitating surface.

To deduce the mechanism behind the Leidenfrost rotation, we focus on the release of vapour from the surface of the levitating rotor. Following the recent work in refs 4, 12, 13, our model is based on the vapour rectification by the underlying surface, which induces a net viscous drag along the azimuthal direction on the levitating dry-ice disc or water film (Fig. 4). We assume that the energy flux across the vapour layer, *q*_in_, occurs by conduction, that is, *q*_in_≈*λ*Δ*T*/*h*, where Δ*T* is the temperature difference across the vapour layer, of thermal conductivity *λ* and thickness *h.* For temperatures above the Leidenfrost point, the energy flux is mainly expended in the phase change of the fuel (the liquid or the ice). This allows us to estimate the speed of evaporation at the rotor surface, *ν*_*n*_≈*q*_in_/*σρ*=*λ*Δ*T*/*σρh*, where *σ* is the latent heat associated with the phase change and *ρ* is the density of the vapour. As depicted in Fig. 4, the vapour stream is rectified by the turbine, causing a net flow along the azimuthal coordinate and downhill along the teeth.

To determine the flow pattern within the vapour layer, we use the hydrodynamic mass and momentum conservation laws for an incompressible fluid, which correspond to the familiar continuity and Navier−Stokes equations. A dimensional analysis reveals that the ratio of inertial to viscous forces acting on a fluid element of vapour can be quantified by the Reynolds numbers Re_*r*_=*ρh*^2^*U*_*r*_/*ηR* and Re_*θ*_=*ρh*^2^*U*_*θ*_/*ηl*, corresponding to the radial and azimuthal components of the flow. Here *U*_*r*_ and *U*_*θ*_ are the typical radial and angular velocities, and *η* is the dynamic viscosity of the vapour. From mass conservation we find *U*_*r*_=(*R*/*h*)*ν*_*n*_, which eventually leads to Re_*r*_≈*λ*Δ*T*/*ησ*. This ratio is of the order 10^−2^ for the material properties and temperature differences of both water and dry-ice loads (see Supplementary Table 1 for a list of physical properties). Noting that *l*=2*πr*/*N*, the azimuthal Reynolds number reads Re_*θ*_=*ρh*^2^*ωN*/2*πη*, where *ω* is the angular velocity of the disc. Using *h*~*H*, we then find Re_*θ*_≈0.2. Therefore, the flow within the vapour layer is dominated by viscous friction. Furthermore, because the vapour layer thickness, *h*, is much smaller than the lateral length scale of the gap, *R*, we can invoke the lubrication approximation of the hydrodynamic equations^14^. The continuity and Navier–Stokes equations are henceforth reduced to


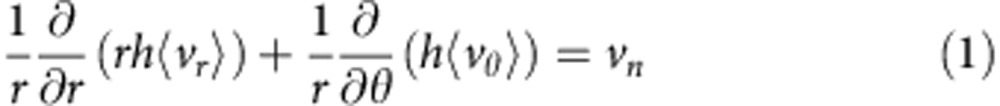



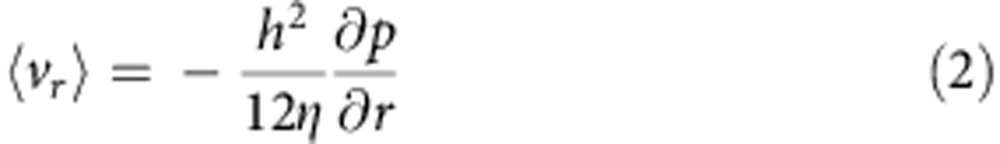


and


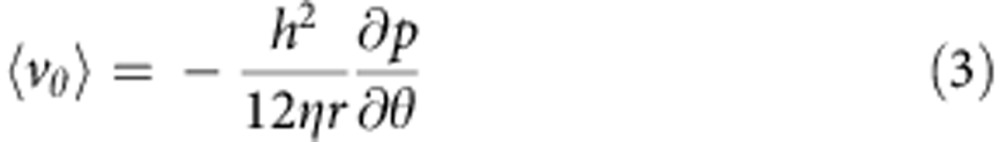


The first equation is the continuity equation averaged over the thickness of the vapour layer, where ‹*v*_*r*_› and ‹*v*_*θ*_› are the local radial and azimuthal components of the velocity field (also averaged over the thickness of the vapour layer). The second and third equations correspond to Darcy’s law, and determine the relation between the local average velocity and the gradient of the pressure field, *p*(*r*,*θ*). Substitution of equations (2) and (3) into equation (1) gives the following second-order partial differential equation for the pressure field:


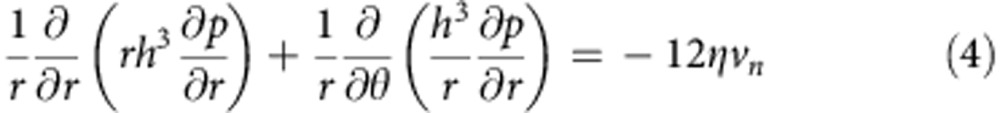


The effect of the underlying tooth pattern enters in the variation of the local thickness, and consequently in the speed of release of the vapour, that is, *h*=*h*(*r*,*θ*) and *v*_*n*_=*v*_*n*_(*h*(*r*,*θ*)). To simplify the mathematical problem, we consider the effect of small local inclination angles, that is, 
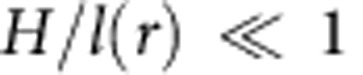
, and focus on the limit where the height of the teeth is small compared with the typical thickness of the vapour layer. The local layer thickness can thus be approximated by


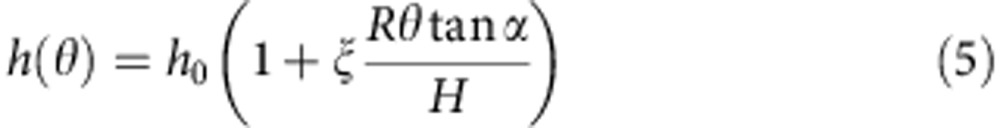


where *h*_0_ is the thickness of the layer for a flat turbine and 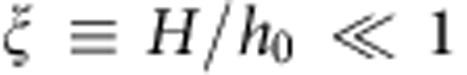. The hydrodynamic equations can be solved perturbatively in powers of *ξ* by linearizing equation (4) and writing


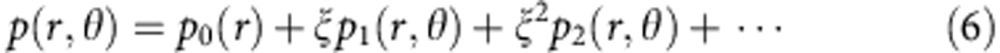


The pressure field then follows by substituting this ansatz into equation (4), solving order by order in *ξ*. The perturbation solution gives the leading order contributions to the flow field in the vapour layer. For a detailed derivation of the solution of the pressure field, we refer the reader to the Supplementary Note 1.

Because of the (approximately) uniform vapour release at the surface of the rotor, the pressure profile decays from the centre of the bottom surface towards the edge. This is captured by the zeroth-order contribution to the pressure,


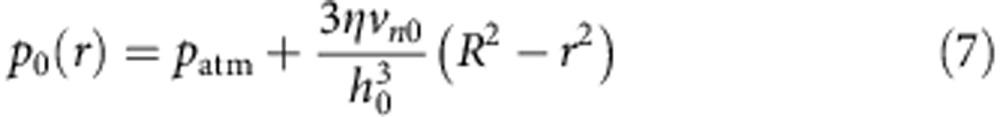


where *v*_*n*0_=*v*_*n*_(*h*_0_). This excess pressure balances the weight of the rotor, leading to levitation, and determines the thickness of the vapour layer *h*_0_ (ref. 4). Because the turbine substrates are not flat, levitation is favoured when the thickness of the vapour layer is larger than the depth of the teeth, thus avoiding contact between the two surfaces. In our experiments, *H* is of the order of hundreds of microns, we thus expect that close to the onset of rotation the vapour layer thickness is of the same order. By setting *h*_0_≈*H*, we obtain a criterion for the critical mass to achieve rotation,


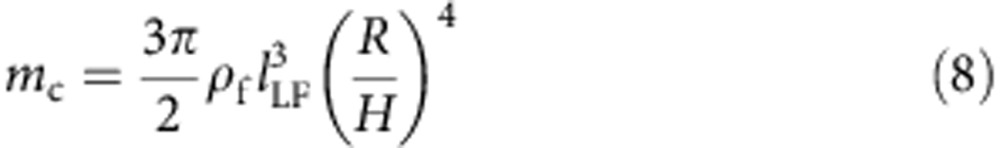


where *l*_LF_≡(*ηλ*Δ*T*/(*σρρ*_f_*g*))^1/3^ is a Leidenfrost length scale characterizing the competition between vapour pressure and the weight of the rotor (of mass density *ρ*_f_).

We now turn our attention to the rotational motion of the loads, which is dominated by the viscous drag acting on the bottom surface of the rotors. From the perturbative solution of the flow within the vapour layer, the average tangential stress acting on the rotor surface along the angular direction is, to leading order in the approximation, 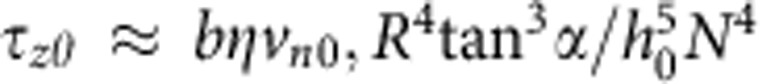, where *b* is a dimensionless constant. This result is consistent with the result of reference 13, which was derived for linear ratchets using scaling arguments and verified numerically. The total torque acting on the disc follows by integrating *rτ*_*zθ*_ over the rotor surface,


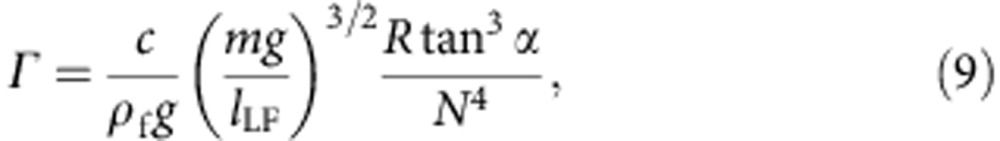


where *c* is a numerical constant. The torque increases with increasing weight because the vapour layer becomes thinner, increasing the local viscous drag. The scaling with increasing radius arises because the drag force has both a longer moment arm and a larger area to act on. Finally, the geometry of the turbine substrate enters in the inclination angle *α*, which determines the degree of rectification and therefore acts to increase the torque. The number of teeth sets the periodicity of the pattern, *L*=2π*R*/*N*, and hampers rectification at large *N*.

## Discussion

**Figure 5:**
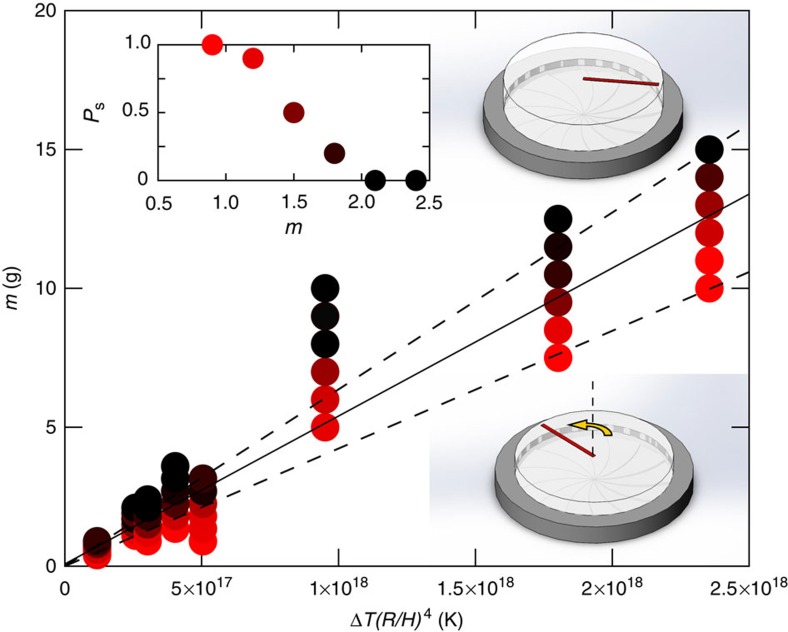
State diagram for the spin-no spin transition of dry-ice rotors. Below a critical mass, discs of dry ice spin on the hot turbines (inset illustrations). The transition is quantified in terms of the probability of a rotating load, *P*_s_, which decreases with increasing mass (for example, the inset figure shows the probabilities *P*_s_ for the six data points corresponding to Δ*T*(*R*/*H*)^4^=3 × 10^17^ K in the main figure). The critical mass, indicated by the solid line, scales linearly with Δ*T*(*R*/H)^4^ as predicted by the theory. The dotted lines correspond to 90% confidence intervals extracted from the probability distributions.

To test our prediction for the critical mass for rotation, equation (8), we carried out experiments over a wide range in the temperature difference, disc radius and average thickness of the turbine teeth (See Supplementary Tables 2 and 3). For each set of experimental conditions (Δ*T, R, H*), we measured the probability of rotation of the disc, *P*_s_(*m*), for a wide range in the mass of the loads, *m* (typically 60 trials). The inset of Fig. 5 shows a typical probability curve, showing the transition to rotation as the mass of the discs is reduced. The experimental state diagram shown in Fig. 5 confirms the scaling of the critical mass, defined as *P*_s_(*m*_c_)=0.5, with Δ*T*(*R*/*H*)^4^, as predicted by equation (8).

**Figure 6:**
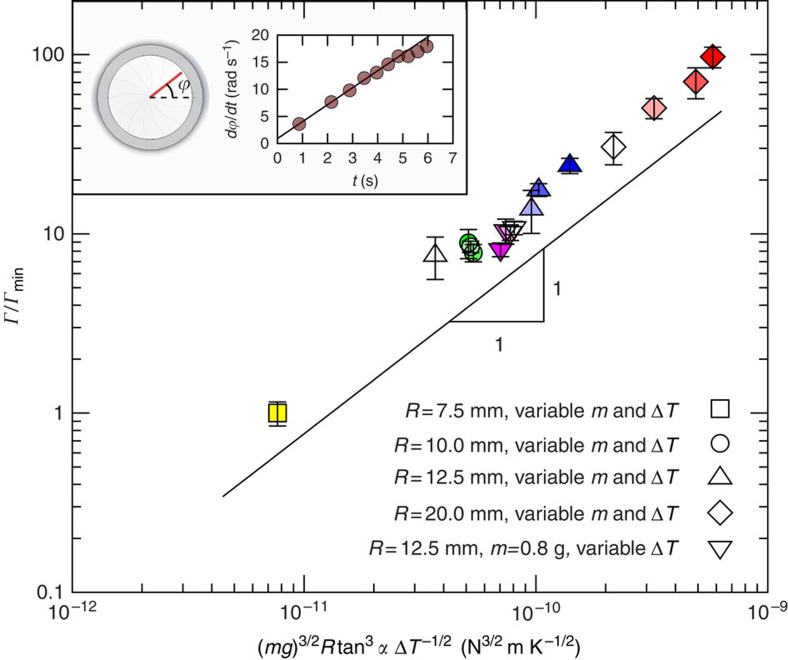
Torque scaling of dry-ice rotors. Tracking the marker on top of spinning discs of dry ice allows us to measure their angular velocity and constant acceleration (inset). The corresponding torque, *Γ*, was deduced from these experiments for a wide range of the disc radii, *R*, temperature difference, Δ*T*, mass, *m* and teeth angle, *α*. The shade intensity within each set of symbols indicates increasing Δ*T*. In the plot, the torque has been normalized by the minimum torque measured, *Γ*_min_=0.0109 μN m. Error bars were calculated using standard error propagation.

To test the theoretical prediction for the torque acting on Leidenfrost rotors, we carried out further experiments measuring the angular acceleration of dry-ice discs of different mass and radii over a range of temperature differences and teeth inclination angles (see inset of Fig. 6 and Supplementary Table 4). We then determined the torque from rigid-body kinematics. The resulting data, shown in Fig. 6 and Supplementary Table 5, shows an excellent agreement (over two decades) with the proposed scaling of equation (9). Moreover, a fit of the data gives a pre-factor within 20% of the theoretical prediction. Such a good agreement suggests that effects arising from inhomogeneities on the turbine substrate and dissipative energy losses are relatively small, thus supporting that the sublimation-based heat engine can be a new approach to energy harvesting.

In our experimental proof-of-concept realization of a sublimation heat engine, the conversion of the latent heat of the phase transition into rotational motion is low in efficiency (~10^−6^). Some of the loss is due to the viscous dissipation within the gap, some is due to the escape of gas along the turbine edge and some is from the evaporation from the top and side faces of the disc. However, a large fraction of the latent heat of the phase transition, either sublimation or thin-film boiling, is used to sustain the levitation of the disc. The total generated power can be written as *P*=*P*_lev_+*P*_rot_, where *P*_lev_=(*πR*^2^*p*_atm_+*mg*)*v*_*n*0_ is the power generated to sustain levitation^15^ and *P*_rot_=Γ*ω* is the power generated by rotation of the disc. The total power should be compared with the rate of energy release due to the phase transition, 
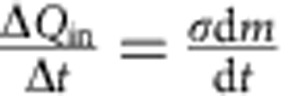
. The speed of release of vapour molecules, *v*_*n*0_, can be found by mass conservation, that is, 
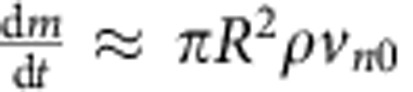
. Using the experimentally measured values for *m*, *R*, d*m*/d*t*, Γ and ω, along with reported values for the physical parameters (see Supplementary Note 1), we find 
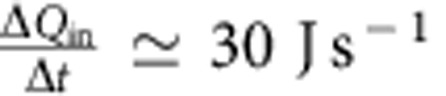
, *P*_lev_≃4.2 J s^−1^ and *P*_rot_≃2 × 10^−5^ J s^−1^. The energy released by the phase transition is therefore dominantly sustaining the levitation, an effect that could be removed by design at the expense of introducing friction within a bearing.

To further demonstrate the feasibility of harvesting thermal energy using the sublimation heat engine, we constructed a simple electric generator. By attaching a frame with eight Neodynium magnets to a dry-ice rotor and lowering a multi-segment induction coil system into close proximity to the rotating assembly, we were able to generate an alternating voltage (see Supplementary Movie 3).

The new concept of a thermal cycle based on either sublimation or thin-film boiling introduced in this paper is appealing because it can lead to new routes for power generation and energy harvesting as we have demonstrated by our proof-of-concept. Future optimized designs of a Leidenfrost-based engine could focus on efficiency using geometries where the gap between the disc and the turbine surface is controlled, where the precise sweep and shape of blades are optimized and where energy losses can be minimized by reducing the escape velocity of the vapour at the edge of the turbine. As supported by our experiments with water, the extension to liquid fuels can be accomplished. Further work in this direction can focus on the design of wicking surfaces that act as fuel-dispensing shafts.

The temperature differences occurring in space and the abundant naturally occurring liquids and ices on planetary bodies^9^ give one example where the transport of fuel is prohibitive, but local conditions can provide all that is needed for a sublimation engine. Given recent progress in reducing the Leidenfrost temperature exploiting superhydrophobic coatings^16^ and low pressures^17^, another potential field of application is in microsystems, where high surface area-to-volume ratios pose significant challenges for any moving part. Here the concept of a motor exploiting the intrinsic low-friction vapour bearing provided by thin-film boiling could have wide applicability.

## Methods

### Dry-ice discs

Dry-ice discs were produced by depositing liquid carbon dioxide (BOC) onto a snowpack dry-ice maker (VWR). The resulting dry-ice snow was shaped into discs using a bespoke pressure mould of variable diameter. Discs were further flattened using a commercial hot plate (VWR VMS-C7) at 150 °C.

### Hot plate

The hot plate used in the experiments consisted of a machined block of aluminium fitted with 2 × 200 W 1/2′′ × 3′′ cartridge heaters (RS Components) and a K-type thermocouple to monitor the temperature. The cartridge heaters were controlled using a Proportional-Integral-Derivative (PID) controller. The hot plate was isolated from the working bench using ceramic pillars.

### Confinement rings

Confinement rings were made from a stock steel bar and were turned on a lathe to have a desired internal diameter and a square cross section of 5 × 5 mm.

### Angular acceleration and torque measurements

The mass and radius of dry-ice discs were measured immediately before each experiment. A small mark was made on the top surface of the discs using a dry-wipe marker to allow visualization of rotational motion. The discs were then placed on the turbine, inside the confinement ring and filmed from above at 50 frames per second using an SVSi MemView high-speed camera. The video files were then analyzed to determine the period of rotation and tracked for the first six rotations. The average angular velocity for each complete rotation was plotted as a function of time. The angular acceleration was then extracted from these measurements. The torque was extracted using data for the angular acceleration assuming rigid-body kinematics.

### Onset of spinning

Experiments were carried out using a single turbine to ensure the consistency of results. The dry-ice disc radius and mass were controlled for each trial using confinement rings of different radii. For each experiment the dry-ice disc was placed on the turbine. A disc was classed as spinning if the disc underwent a full rotation within the first 10 s of being placed on the turbine and sustained the rotational motion for at least five revolutions.

### Turbines

Turbines were manufactured using standard computer numerical control machining from a thin sheet of aluminium.

### Electromagnetic generator

The electromagnetic generator was manufactured by laser cutting two pieces of 3-mm thick medium-density fibreboard and laminating them together to form an eight-lobed commutator. Each lobe was fitted with a Neodynium magnet. The commutator was fixed to the top surface of a dry-ice disc using three small tacks. A stator was made by winding 0.15-mm diameter varnished copper wire into 8 cylindrical coils with a core diameter of 7 mm, an external diameter of 27 mm and length 12 mm, with approximately 4,000 turns per coil. The coils were then laid flat into the lobe pattern to match the commutator and fixed into place using SampleKwick Fast cure acrylic (20-3560). Visualization of the electric signal was performed using a standard oscilloscope.

## Author contributions

The study was jointly conceived, developed and designed. K.S. designed and produced the turbines. G.G.W. carried out the experiments. G.G.W. and R.L.-A. analyzed the data. R.L.-A. developed the theoretical model. G.G.W. designed the proof-of-concept engine. R.L.-A. and G.M. wrote the paper with contributions from G.G.W. and K.S.

## Additional information

**How to cite this article:** Wells, G. G. *et al*. A sublimation heat engine. *Nat. Commun.* 6:6390 doi: 10.1038/ncomms7390 (2015).

## Supplementary Material

Supplementary Tables and Supplementary Note Supplementary Tables 1-5, Supplementary Note 1

Supplementary Movie 1 Rotation of a dry-ice block on a hot turbine-like surface via the Leidenfrost effect.

Supplementary Movie 2 Rotation of an aluminium plate supported by a water droplet on a hot turbine-like surface via the Leidenfrost effect.

Supplementary Movie 3 Electromagnetic generator powered by a sublimation heat engine.
