# Supplementary material for: A sublimation heat engine
Source: Nat Commun. 2015 Mar 3;6:6390. doi: 10.1038/ncomms7390 (PMC4366496; doi:10.1038/ncomms7390)
Supplement: Supplementary Tables and Supplementary Note — Supplementary Tables 1-5, Supplementary Note 1 [file ncomms7390-s1.pdf]

Supplementary Tables

ρ (kg m^{-3})	ρ_{ice} (kg m^{-3})	λ (W m^{-1} K^{-1})	η (μPa s)	ΔH (kJ kg^{-1})
1.2	1118	0.029	22	598

Supplementary Table 1. Physical properties of CO_2 as reported in Baier *et al.*, *Phys. Rev. E*, **87,** 021001, (2013), except for ρ_{ice}, which is the average value measured in the present experiments.

**Disc radius, *R* (± 1mm)**	**Hotplate temperature, T_h (± 5°C)**	**Tooth thickness, *H* (± 10 μm)**
7.5	500	165
10	500	199
12.5	500, 400, 300	212
15	500	227
18	500	232
20	500	229

Supplementary Table 2. Radius, hotplate temperature and tooth thickness for data shown in Fig. 5.

$\Delta T(R/H)^4$ = 1.18x10^{17}			$\Delta T(R/H)^4$ = 2.58 x10^{17}			$\Delta T(R/H)^4$ = 5.04 x10^{17}			$\Delta T(R/H)^4$ = 4.03 x10^{17}		
m(±0.05 g)	P_s	δP_s	*m*(±0.10 g)	P_s	δP_s	*m*(±0.23 g)	P_s	δP_s	*m*(±0.23 g)	P_s	δP_s
0.40	0.90	0.10	1.10	1.00	0.00	0.90	1.00	0.00	1.35	1.00	0.00
0.50	1.00	0.00	1.30	0.90	0.10	1.35	0.91	0.10	1.80	0.80	0.13
0.60	0.80	0.13	1.50	0.50	0.17	1.80	0.90	0.10	2.25	0.30	0.15
0.70	0.80	0.13	1.70	0.00	0.00	2.25	0.70	0.15	2.70	0.10	0.10
0.80	0.40	0.16	1.90	0.30	0.15	2.70	0.10	0.10	3.15	0.00	0.00
0.90	0.20	0.13	2.10	0.00	0.00	3.15	0.20	0.13	3.60	0.00	0.00
$\Delta T(R/H)^4$ = 3.03 x10^{17}			$\Delta T(R/H)^4$ = 9.51 x10^{17}			$\Delta T(R/H)^4$ = 1.80x10^{18}			$\Delta T(R/H)^4$ = 2.35 x10^{18}		
m(±0.15 g)	P_s	δP_s	*m*(±0.5 g)	P_s	δP_s	*m*(±0.5 g)	P_s	δP_s	*m*(±0.5 g)	P_s	δP_s
0.90	1.00	0.00	5.0	0.90	0.10	7.50	1.00	0.00	10.00	1.00	0.00
1.20	0.90	0.10	6.0	0.82	0.13	8.50	0.90	0.10	11.00	1.00	0.00
1.50	0.50	0.17	7.0	0.60	0.16	9.50	0.50	0.17	12.00	0.80	0.13
1.80	0.20	0.13	8.0	0.00	0.00	10.50	0.20	0.13	13.00	0.60	0.16
2.10	0.00	0.00	9.0	0.09	0.10	11.50	0.10	0.10	14.00	0.30	0.15
2.40	0.00	0.00	10.0	0.00	0.00	12.50	0.00	0.00	15.00	0.00	0.00

Supplementary Table 3. Probability of spinning and standard error for data shown in Fig. 5.

R (± 1 mm)	20	12	12	10	7.5
α (± 1°)	2.25	3.40	3.40	3.62	4.14 (± 2°)
T_h (± 5 °C) 🡻	mass (± 0.1g)	mass (± 0.1g)	mass (± 0.1g)	mass (± 0.1g)	mass (± 0.1g)
350	2.58	0.77	0.79	-	-
	2.42	0.81	0.76	-	-
	2.49	0.78	0.74	-	-
	2.54	0.85	0.79	-	-
	2.56	0.74	0.79	-	-
400	3.46	0.91	0.79	0.63	-
	3.42	0.94	0.80	0.59	-
	3.32	0.88	0.79	0.60	-
	3.43	0.89	0.73	0.62	-
	3.45	0.94	0.80	0.66	-
450	4.69	1.00	0.73	0.61	-
	4.59	0.98	0.80	0.70	-
	4.64	0.96	0.81	0.67	-
	4.62	0.94	0.80	0.65	-
	4.63	1.02	0.80	0.66	-
500	5.45	1.22	0.79	0.66	0.20
	5.21	1.28	0.80	0.69	0.19
	5.28	1.29	0.80	0.64	0.19
	4.03	1.30	0.79	0.66	0.19
	5.29	1.18	0.75	0.64	0.17

Supplementary Table 4. Radius, tooth angle, hotplate temperature and mass for data shown in Fig. 6.

Average m (g)	R (mm)	ΔT (°C)	α (°)	Γ (x10^6) (N m)
5.30	20	576	2.25	1.06 ± 0.14
4.63	20	526	2.25	0.77 ± 0.15
3.42	20	476	2.25	0.55 ± 0.07
2.52	20	426	2.25	0.33 ± 0.07
1.25	12.5	576	3.40	0.26 ± 0.03
0.98	12.5	526	3.40	0.19 ± 0.02
0.91	12.5	476	3.40	0.15 ± 0.04
0.47	12.5	426	3.40	0.08 ± 0.02
0.79	12.5	576	3.40	0.09 ± 0.01
0.79	12.5	526	3.40	0.11 ± 0.02
0.78	12.5	476	3.40	0.11 ± 0.01
0.77	12.5	426	3.40	0.12 ± 0.01
0.66	10	576	3.62	0.10 ± 0.02
0.66	10	526	3.62	0.09 ± 0.01
0.62	10	476	3.62	0.090 ± 0.007
0.19	7.5	576	4.14	0.010 ± 0.002

Supplementary Table 5. Torque data for each data point shown in Fig. 6

Supplementary Notes

Supplementary Note 1. Hydrodynamic Model

We consider rotors of of radius R, and thickness h_{f}, which are deposited on turbine-like surfaces of the same radius. Using cylindrical coordinates, both objects are concentric, and their main axis is parallel the z axis. The turbine is patterned with N triangular grooves that extend from its centre along the radial coordinate r, and which slope down with increasing angular coordinate θ. The depth of the grooves H is constant, while their local span $l(r)$ is fixed by

$$l(r) = \frac{2\pi r}{N}, \tag{1}$$

where N is the number of grooves. Because the thickness is kept constant, the local slope becomes larger at the centre of the disc, and obeys

$$\frac{H}{l(r)} = \left(\frac{R}{r}\right) \tan\alpha, \tag{2}$$

where $\tan\alpha \equiv H/L$ is the slope at the rim of the disc, where the grooves reach their maximum span, i.e., $L = l(R)$.

The temperature difference between the surface of the rotor and the heated surface, ΔT, drives the release of vapour which accumulates in the gap between the turbine and the rotating disc. We assume that the relaxation of the temperature profile in the vapour layer is instantaneous. If the local thickness of the gap is denoted by h, and we assume that the energy flux is completely expended in the transfer of solid to gas molecules, then the speed of release of gas molecules into the gap is

$$v_n \approx \frac{\lambda}{\rho \Delta H} \frac{\Delta T}{h}, \tag{3}$$

where λ is the thermal conductivity of the vapour, ρ its density and ΔH the latent heat of the phase change.

In steady state, and assuming that the vapour is incompressible, the flow is governed by the continuity and Navier-Stokes equations,

$$\nabla \cdot \mathbf{v} = 0, \tag{4}$$

and

$$\rho(\mathbf{v} \cdot \nabla)\mathbf{v} = -\nabla p + \eta \nabla^2 \mathbf{v} + \rho \mathbf{g}, \tag{5}$$

where $\mathbf{v}(r, \theta, z)$ and $p(r, \theta, z)$ are the velocity and pressure fields, η is the viscosity of the vapour, and $\mathbf{g}$ is the acceleration due to gravity.

A dimensional analysis of Eq. (5) indicates that the relative magnitude between inertial and viscous forces can be expressed in terms of the Reynolds number

$$\mathrm{Re} \equiv \frac{\rho h^2 U}{R \eta}. \tag{6}$$

From mass conservation we can find the scaling for the typical radial velocity U, i.e.,

$$\pi R h U \sim \pi R^2 v_n, \tag{7}$$

For example, using the physical parameters reported in Supplementary Table 1 for CO_2 we find

$$\mathrm{Re} \sim \frac{\lambda \Delta T}{\eta \Delta H} \approx 0.01, \tag{8}$$

indicating that inertial effects are negligible. We can further simplify the problem by noting that the aspect ratio

H/R and the inclination $\tan\alpha$ are small compared to unity. This allows us to invoke the lubrication approximation, from which Eq. (5) simplifies to

$$\eta \frac{\partial^2 \mathbf{v}_\parallel}{\partial z^2} = \nabla_\parallel p, \tag{9}$$

where the pressure field $p(r,\theta)$ depends on r and θ only, $\mathbf{v}_\parallel \equiv \hat{\mathbf{r}} v_r + \hat{\boldsymbol{\theta}} v_\theta$ and $\nabla_\parallel \equiv \hat{\mathbf{r}}\partial_r + \hat{\boldsymbol{\theta}} r^{-1}\partial_\theta$. Integrating Eq. (9) twice with respect to z and imposing the stick boundary condition at both the bottom and top surfaces we find that the in-plane velocity components obey

$$v_r(r,\theta,z) = \frac{z(z-h)}{2\eta}\frac{\partial p}{\partial r}, \tag{10}$$

and

$$v_\theta(r,\theta,z) = \frac{z(z-h)}{2\eta r}\frac{\partial p}{\partial \theta}. \tag{11}$$

Eqs. (10) and (11) can now be substituted into Eq. (4) to eliminate the velocity field. Integrating in z from $z=0$ to $z=h$, and imposing the boundary conditions $v_z(z=0)=0$ and $v_z(z=h)=-v_n$ gives

$$\frac{1}{r}\frac{\partial}{\partial r}\left(r h^3 \frac{\partial p}{\partial r}\right) + \frac{1}{r}\frac{\partial}{\partial \theta}\left(\frac{h^3}{r}\frac{\partial p}{\partial \theta}\right) = -12\eta v_n. \tag{12}$$

Our aim is to solve Eq. (12) to obtain the pressure profile $p(r,\theta)$. Knowing the pressure allows us to calculate the levitating force. This determines the thickness at which the film levitates and the viscous stress on the surface of the rotor.

In order to estimate the flow in the vapour layer, we consider the effect of small surface deformations relative to the thickness of the film that lies between the tips of the grooves and the rotor surface. If the thickness of the reference film is h_0 and the characteristic thickness of the grooves is H, then we can write

$$h(r,\theta) = h_0\left(1 + \epsilon f(r,\theta)\right), \tag{13}$$

where $f(r,\theta)$ controls the shape of the deformation and $\epsilon \equiv H/h \ll 1$. Next, we expand the pressure field in powers of ϵ:

$$p(r,\theta) = p_0(r) + \epsilon p_1(r,\theta) + \epsilon^2 p_2(r,\theta) + \cdots. \tag{14}$$

Similarly, for v_n we have

$$v_n(r,\theta) = v_{n0}\left(1 - \epsilon f(r,\theta) + \epsilon^2 f^2(r,\theta) + \cdots\right) \tag{15}$$

where $v_{n0} \equiv \lambda \Delta T/\rho \Delta H h_0$.
Substituting Eqs. (13), (14) and (15) into Eq. (12) we find, to order ϵ^0:

$$\frac{1}{r}\frac{d}{dr}\left(r\frac{dp_0}{dr}\right) = -\frac{12\eta v_{n0}}{h_0^3}, \tag{16}$$

to order ϵ^1:

$$\frac{1}{r}\frac{\partial}{\partial r}\left(r\frac{\partial p_1}{\partial r}\right) + \frac{1}{r^2}\frac{\partial^2 p_1}{\partial \theta^2} = -\frac{3}{r}\frac{\partial}{\partial r}\left(r f \frac{dp_0}{dr}\right) + \frac{12\eta v_{n0}}{h_0^3} f, \tag{17}$$

to order ϵ^2:

$$\frac{1}{r}\frac{\partial}{\partial r}\left(r\frac{\partial p_2}{\partial r}\right) + \frac{1}{r^2}\frac{\partial^2 p_2}{\partial \theta^2} = -\frac{3}{r}\left[\frac{\partial}{\partial r}\left(r f^2 \frac{dp_0}{dr}\right) + \frac{\partial}{\partial r}\left(r f \frac{\partial p_1}{\partial r}\right) + \frac{1}{r}\frac{\partial}{\partial \theta}\left(f \frac{\partial p_1}{\partial \theta}\right)\right] - \frac{12\eta v_{n0}}{h_0^3} f^2, \tag{18}$$

etc.

Our aim is to solve for the different contributions to the pressure field, order by order in ϵ, subject to the boundary conditions

$$p_0(R) = p_{\text{atm}}, \quad \frac{dp_0}{dr}(0) = 0, \tag{19}$$

and

$$p_i(R,\theta) = 0, \quad p_i(r,0) = p_i(r,2\pi/N), \quad i = \{1,2,\ldots\}, \tag{20}$$

where p_{atm} is the atmospheric pressure.

Equation (16) can be integrated directly to give the pressure distribution across a homogeneous gap. Using the boundary conditions (19) we find

$$p_0(r) = p_{\text{atm}} + \frac{3\eta v_{n0}}{h_0^3}(R^2 - r^2). \tag{21}$$

The pressure thus decreases with the radial coordinate, and the maximum pressure is proportional to R^2.

From Eq. (17) we notice that to make further progress we need to specify the function $f(r,\theta)$. We expect that the dominant effect of the ratchet on the flow is caused by the gently sloping face of each groove. For our ratchets, we therefore have

$$f(\theta) = \frac{R}{H}\tan\alpha\theta. \tag{22}$$

We can now substitute the expressions for $p_0(r)$ and $f(r,\theta)$ into Eq. (17) to obtain a PDE for $p_1(r,\theta)$, i.e.,

$$\frac{\partial}{\partial r}\left(r\frac{\partial p_1}{\partial r}\right) + \frac{1}{r}\frac{\partial^2 p_1}{\partial\theta^2} = \frac{48\eta v_{n0}\tan\alpha}{h_0^3 H} Rr\theta. \tag{23}$$

This equation admits the solution

$$p_1 = r^2 g_1(\theta). \tag{24}$$

Substituting Eq. (24) into Eq (23) leads to

$$\frac{d^2 g_1}{d\theta^2} + 4g_1(\theta) = \frac{48\eta v_{n0}\tan\alpha}{h_0^3 H} R\theta, \tag{25}$$

whose solution is

$$g_1(\theta) = c_2\sin(2\theta) + c_3\cos(2\theta) + 12\frac{\eta v_{n0}\tan\alpha}{h_0^3 H} R\theta. \tag{26}$$

Using the boundary conditions (20), we find

$$p_1(r,\theta) = 12\frac{\eta v_{n0}\tan\alpha}{h_0^3 H}\left(\theta - \frac{2\pi}{N}\frac{\sin(2\theta)}{\sin\left(\frac{4\pi}{N}\right)}\right) Rr^2. \tag{27}$$

Note that this solution does not satisfy the boundary condition $p_1(R,\theta) = 0$. This is a result of the particular choice for the ratchet shape which misses the periodicity of the pattern. This will give rise to a small error in the radial flux at the disc boundary $\epsilon\partial_r p_1(R,\theta)/\partial_r p_0(R) \sim \epsilon$. However, the pre-factor in Eq. (27) should retain the same scaling as the approximation for the surface shape improves. On this basis, results following from Eq. (27) should be qualitatively correct.

The second and third-order contributions p_2 and p_3 have the form

$$p_2(r,\theta) = r^2 g_2(\theta), \tag{28}$$

and

$$p_3(r,\theta) = r^2 g_3(\theta), \tag{29}$$

However, the expression for g_2 and g_3 are lengthy and we thus omit its presentation here.

The excess pressure in the gap exerts a net upwards force on the rotor, which makes it levitate. This force is

$$\mathbf{F} = N \int_0^R r dr \int_0^{2\pi/N} p(r,\theta) d\theta \hat{\mathbf{z}}. \tag{30}$$

which can be written in powers of ϵ as

$$\mathbf{F} = \mathbf{F}_0 + \epsilon \mathbf{F}_1 + \epsilon^2 \mathbf{F}_2 \cdots . \tag{31}$$

For $N \gg 1$, which corresponds to the limit where our analysis is applicable, the first non-vanishing term in ϵ is

$$F_2 \sim \frac{F_0}{N^2}, \tag{32}$$

and is therefore negligible.

Hence

$$\mathbf{F} \approx \frac{3\pi \eta v_{n0} R^4}{2h_0^3} \hat{\mathbf{z}}. \tag{33}$$

For levitation to occur, this force must be balanced by the weight of the rotor, $m\mathbf{g} = -\pi R^2 h_\mathrm{f} \rho_\mathrm{f} g \hat{\mathbf{z}}$, which sets the thickness of the vapour layer. Noting that

$$v_{n0} = \frac{\lambda}{\rho \Delta H} \frac{\Delta T}{h_0}, \tag{34}$$

we arrive at

$$h_0 = \left(\frac{3}{2}\right)^{1/4} \left(\frac{\eta \lambda \Delta T}{\rho \Delta H \rho_\mathrm{f} g}\right)^{1/4} \frac{R^{1/2}}{h_\mathrm{f}^{1/4}}. \tag{35}$$

The second term in brackets can be written in terms of a "Leidenfrost length scale" l_LF characterising the competition between gravity and gas pressure, i.e.,

$$l_\mathrm{LF} \equiv \left(\frac{\eta \lambda \Delta T}{\rho \Delta H \rho_\mathrm{f} g}\right)^{1/3}, \tag{36}$$

whereby

$$h_0 = \left(\frac{3}{2}\right)^{1/4} l_\mathrm{LF}^{3/4} \frac{R^{1/2}}{h_\mathrm{f}^{1/4}}. \tag{37}$$

Using the physical parameters reported in Supplementary Table 1 we find $l_\mathrm{LF} \sim 10$ μm. For a rotor of radius $R = 2.0$ cm and thickness $h_\mathrm{f} = 5$ mm we find that the film thickness if of the order of 10^2 μm.

For the rotor to spin, the vapour layer must be of the same order, or larger, than the thickness of the corrugation. Setting $h_0 = H$ in Eq. (37) gives

$$mg = \frac{3\pi}{2} \left(\frac{\eta \lambda}{\rho \Delta H}\right) \frac{\Delta T R^4}{H^4}. \tag{38}$$

Therefore, the maximum weight for a spinning disc scales as ΔT, R^4 and H^{-4}.

The local tangential stress (in the angular direction) is

$$\tau_{\theta z} = \eta \frac{\partial v_\theta}{\partial z} = \frac{2z-h}{2r}\frac{\partial p}{\partial \theta}, \tag{39}$$

We estimate the average stress on the rotor, i.e.,

$$\hat{\tau}_{\theta z} = \frac{N}{\pi R^2}\int_0^R r dr \int_0^{2\pi/N} \tau_{\theta z} d\theta. \tag{40}$$

Taking into consideration the approximation of the shape of the ratchet, we consider only terms which are odd in the slope. The first non-vanishing contribution satisfying this symmetry is of order ϵ^3, i.e.,

$$\hat{\tau}_{\theta z} = -\frac{224\pi^4}{15}\frac{\eta v_{n0} R^4 \tan^3\alpha}{h_0^2 H^3 N^4}\epsilon^3, \tag{41}$$

which simplifies to

$$\hat{\tau}_{\theta z} = -\frac{14}{15}\frac{\eta v_{n0} L^4 \tan^3\alpha}{h_0^5}. \tag{42}$$

Recalling the scaling for v_{n0} and h_0 we obtain

$$\hat{\tau}_{\theta z} \sim \frac{LH^3}{R^3} \tag{43}$$

which agrees with the scaling obtained using numerical simulations in Baier et al. (*Phys. Rev. E*, 87 021001 (2013)).

The torque acting on the rotor is

$$\mathbf{T} = -N\int_0^R r^2 dr \int_0^{2\pi/N} \tau_{\theta z} d\theta \hat{\mathbf{z}}. \tag{44}$$

Again, the first non-vanishing, $\tan\alpha$-odd, contribution is of order ϵ^3 and reads

$$\mathbf{T} = \frac{56\pi^5}{5}\frac{\eta v_{n0} R^7 \tan^3\alpha}{h_0^5 N^4}\hat{\mathbf{z}}. \tag{45}$$

Substituting the expression for v_{n0} we obtain

$$\mathbf{T} = \left(\frac{56\pi^5}{5}\right)\frac{\eta\lambda}{\rho\Delta H}\frac{\Delta T R^7 \tan^3\alpha}{h_0^6 N^4}\hat{\mathbf{z}}. \tag{46}$$

The torque can be expressed in terms of the total weight supported by the vapour layer (including any loads):

$$\mathbf{T} = c\left(\frac{\rho\Delta H}{\eta\lambda}\right)^{1/2}\frac{(mg)^{3/2} R \tan^3\alpha}{\Delta T^{1/2} N^4}\hat{\mathbf{z}}, \tag{47}$$

where $c \equiv (56\pi^5/5)(2/3\pi)^{3/2}$.
